# Heat shock protein 70 could enhance porcine epidemic diarrhoea virus replication by interacting with membrane proteins

**DOI:** 10.1186/s13567-021-01006-9

**Published:** 2021-10-30

**Authors:** Jae-Yeon Park, Jihoon Ryu, Jung-Eun Park, Eui-Ju Hong, Hyun-Jin Shin

**Affiliations:** 1 College of Veterinary Medicine, Chungnam National University, Daejeon, 13434 Republic of Korea; 2 Research Institute of Veterinary Medicine, Chungnam National University, Daejeon, 13434 Republic of Korea

**Keywords:** Porcine epidemic diarrhoea virus, heat shock protein 7, replication

## Abstract

In this study, we investigated the role of heat shock protein 70 (HSP70) in porcine epidemic diarrhoea virus (PEDV) replication. We found that PEDV infection induced strong HSP70 overexpression in the very early stage of infection. We also confirmed that HSP70 overexpression increased the speed of PEDV replication, resulting in the generation of more virions. In contrast, knockout of HSP70 in cells significantly downregulated PEDV protein expression, resulting in a significant reduction in PEDV replication. Most importantly, we confirmed that among the structural proteins of PEDV, membrane (M) proteins have this important role. We found that membrane proteins control cellular HSP70 expression in PEDV-infected cells. We confirmed HSP70/M complex formation by both immunoprecipitation and immunofluorescence assays. Additionally, PEDV M overexpression induced strong HSP70 expression. All our results clearly confirmed that in PEDV-infected cells, the M protein plays a very important role in PEDV replication in collaboration with HSP70.

## Introduction

Viral replication is dependent on host cellular factors [1], several of which are necessary for viral replication [2]. In virus-infected cells, proteotoxic stress is strongly induced by viral protein synthesis [3], which has a negative effect on viral replication [4]. Cells respond to stress through adaptive changes that either limit or repair damage, thereby preventing cell death [5]. Heat shock protein 70 (HSP70) is an important protein that promotes cellular homeostasis during metabolism [6]. HSP70, a member of the heat shock protein family, is involved in various cellular processes through its role as a molecular chaperone [7]. In particular, it helps refold proteins that are misfolded or aggregated due to cell stress [8]. HSP70 family members contain two domains: the N-terminal ATPase domain and the C-terminal substrate-binding domain [9]. ATP hydrolysis induces a conformational change in HSP70, triggering substrate folding and release [10].

Interaction with chaperones, such as heat shock proteins, is important for viral replication [4, 11]. Interactions between viral proteins and heat shock proteins have been reported in several viruses, such as tombusvirus, reovirus, adenovirus, polyomavirus, dengue virus, and influenza virus. [12–15]. However, not all viruses interact with host heat shock proteins. For example, HIV-1 viral protein R and the influenza A virus ribonucleoprotein negatively interact with HSP70 [16, 17].

Porcine epidemic diarrhoea virus (PEDV) infects swine intestines, causing enteric diseases along with vomiting, diarrhoea, dehydration, and high mortality in suckling piglets [18, 19]. PEDV is a positive single-stranded RNA virus with an ~ 28 kilobase genome containing a 5′ cap and a 3′ polyadenylated tail. The genome comprises seven open reading frames (ORFs), including large polyproteins ORF1a, ORF1b, and ORF2–6, which encode 16 nonstructural proteins and four structural proteins [spike (S), envelope (E), membrane (M), nucleocapsid (N)], and accessory (ORF3) proteins [20, 21]. The role of each structural protein has been well studied. The PEDV N protein interacts with nucleolar phosphoprotein nucleophosmin (NPM1), which promotes PEDV growth [22]. The authors noted that PEDV replicates in the cytoplasm, but the PEDV N protein is associated with nucleolar components other than NPM1. NPM1 overexpression induced PEDV replication, whereas NPM1 knockdown reduced PEDV replication. PEDV ORF3 interacts with cellular vacuolar protein sorting-associated protein 36 (VPS36) to inhibit PEDV replication [23].

The PEDV N protein prolongs the S-phase of the cell cycle, induces endoplasmic reticulum stress (ER stress), and upregulates interleukin-8 (IL-8) expression. PEDV ORF3 prolongs S-phase and facilitates the formation of vesicles that promote the proliferation of PEDV [24]. PEDV ORF3 induces viral replication by inhibiting apoptosis in the host [25] and causes ER stress, facilitating autophagy [26]. In addition, the PEDV E protein causes ER stress and upregulates IL-8 expression. The PEDV S protein is the main receptor-binding protein that interacts with host cell receptors [27]. There have been many studies on virulence factors in PEDV [28–30], but there are few reports on their correlation with host factors. In this study, we confirmed that HSP70 is important for PEDV replication, viral protein synthesis, and assembly.

## Materials and methods

### Cells and viruses

African green monkey kidney cell lines (Vero) were cultured in Minimum Essential Media (MEM) supplemented with 10 mM HEPES (Gibco, USA) and 10% antibiotic–antimycotic (Gibco, USA) foetal bovine serum (Gibco, USA) at 37 °C with 5% CO_2_. 239 T cell lines were cultured in Dulbecco’s modified Eagle’s medium (DMEM) supplemented with 1× antibiotic–antimycotic and 10% foetal bovine serum at 37 °C with 5% CO_2._ IPEC J2 cell lines were cultured in Dulbecco’s Modified Eagle Medium: Nutrient Mixture F-12 (DMEM/F12) supplemented with 1× Insulin-Transferrin-Selenium-G Supplement (100×), 1× Antibiotic–Antimycotic, 10% foetal bovine serum, at 37 °C with 5% CO_2_. In this study, we used the PEDV strain PED-CUP-B2014 [31]. Viral titres were determined by TCID_50_ and plaque assays [32].

### Plasmids

The pCAGGS PEDV structure protein plasmid encoding the PEDV E, M, N and S proteins with a c-Myc fusion at its C-terminus was constructed with the pCAGGS vector. The cDNA for the PED-CUP-B2014. cDNA corresponding to the PEDV structure protein coding region was amplified with specific primers (Table 1). The pCAGGS HSP70 plasmid encoding Chlorocebus sabaeus HSP70 (NCBI Reference Sequence: XM_007973082.1) with a flag fusion at its C-terminus was constructed by the pCAGGS vector. The pLenti-HSP70 plasmid encoding the HSP70.

**Table 1:** **Primer used in this study.**

Primer	Sequence (5′-3′)
PEDV E F	*CTCGAG*GCCACC**ATG**CTACAATTAGTGAATGATAATG
PEDV E R	*AGATCT*TACGTCAATAACAGTACTGGGGAGGG
PEDV M F	*CTCGAG*GCCACC**ATG**TCTAACGGTTCTATTCCCGTTG
PEDV M R	*AGATCT*GACTAAATGAAGCACTTTCTCACTATC
PEDV N F	*CTCGAG*GCCACC**ATG**GCTTCTGTCAGCTTTCAGGATC
PEDV N R	*GGATCC*ATTTCCTGTATCGAAGATCTCGTTG
PEDV S F	*CTCGAG*GCCACC**ATG**AAGTCTTTAAATTACTTCTGG
PEDV S R	*GGTACC*CTGCACGTGGACCTTTTCAAAAAC
HSP70 F	*GATCTC*GCCACC**ATG**GCCAAAGCCGCGGCGATC
HSP70 R	*CTCGAG*ATCTACCTCCTCAATGGTGGGGC
qHSP70 F	AACGCCCTGGAGTCATACGC
qHSP70 R	TCTTCTTGTCGGCCTCGCTG
qPEDV M F	CGTACAGGTAAGTCAATTAC
qPEDV M R	GATGAAGCATTGACTGAA
qGAPDH F	CCTTCCGTGTCCCCACTGCCAAC
qGAPDH R	GACGCCTGCTTCACCACCTTCT

Kozak sequence is underlined. Start codon is bolded. Italic sequence denotes restriction enzyme site.

### Antibodies

Anti-HSP70 antibody (Enzo Life Science, ADI-SPA-811-D), anti-c-Myc antibody (Santa Cruz, sc-40, sc-789), anti-flag antibody (Santa Cruz, sc-807) anti-β-actin (Santa Cruz, sc-47778) and mouse anti-PEDV were made in our laboratory (immunized inactivated PEDV in mouse).

### Heat shock and quercetin treatment to Vero cells

To increase the expression of endogenous HSP70, Vero cells were heated at 45 ℃ for 20 min in a heating block and then transferred to a 37 °C CO_2_ incubator. Vero cells were treated with 100 μM quercetin (dissolved in DMSO and made a stock solution at 100 mM) for 12 h and then washed three times with PBS. In a preliminary study, 100 μM quercetin was the best concentration in VERO cells after 12 h of incubation and no more activity afterwards (data not shown).

### Real-time PCR assay

Vero cells were seeded in 6-well plates. The cells were washed with PBS and lysed in RiboEX Total RNA (GeneAll, Korea), and reverse transcription was performed using BioFACT™ 2× RT Pre-Mix (BioFACT™, Korea) amplified with random hexamer (Thermo Fisher, USA). Amplification was carried out in a 20 μL reaction mixture containing 10 μL TOPreal™ qPCR 2× premix (Enzynomics, Korea), 0.2 μM concentration of each primer (Table 1), and 1 μL cDNA. The reaction procedure was 95 °C for 5 min, followed by 40 cycles at 95 °C for 30 s, 58 °C 30 s and 72 °C 30 s. The relative mRNA expression level was normalized to the housekeeping gene GAPDH. The relative transcript levels were analysed using the ΔΔ Ct method.

### Confocal fluorescence microscopy

We followed the general manual for confocal microscopy. Briefly, Vero cells were seeded in 12-well plates. The cells were treated with PED-CUP-2014B (MOI = 1). At 18 h post-infection, the cells were fixed with 4% paraformaldehyde for 15 min. Following fixation three times washed in PBS, the cells were then permeabilized with 0.25% Triton X-100 for 10 min and blocked in PBS containing 2% BSA for 1 h at room temperature. After three washes with PBS, the coverslips were incubated with primary antibodies in PBS containing 2% BSA at 4 °C (overnight). After being washed with PBS, the coverslips were incubated with Alexa Fluor 488 (Invitrogen, A32723) and Alexa Fluor 594 (Invitrogen, A32740) for 2 h at room temperature. After washing with PBS, the nuclei were stained on Hoechst 33258 (Thermo Fisher, 62249). After staining for 15 min, the cells were washed with PBS and mounted onto microscope slides. Fluorescence signals were observed under confocal microscopy. Transfected samples were also subjected to the same procedure.

### Western blots

The cells were washed with cold PBS and lysed in RIPA buffer (Thermo Fisher, USA) containing protease inhibitor cocktail (Sigma Aldrich, Germany) for 30 min at 4 °C, and then the supernatant was collected. The protein concentration was determined with a BCA protein assay kit (Thermo Fisher, USA). SDS–PAGE and Western blotting were carried out using standard methods. Briefly, equivalent amounts of protein were separated on polyacrylamide-tricine gels (8–12% polyacrylamide). After SDS–PAGE, the gels were transferred onto 0.45 μm polyvinylidene fluoride (PVDF) membranes (Millipore, USA) followed by blocking with 5% BSA in TBST (TBS with 0.1% Tween 20) for 1 h at room temperature. The membrane was incubated with primary antibody at 4 °C overnight. After being washed with TBST, the membrane was incubated with HRP-tagged anti-rabbit IgG and anti-mouse IgG (1:10 000 dilution) for 2 h at room temperature. The images were observed with ECL solution (SuperSignal West Femto Maximum Sensitivity Substrate 34095) using an ATTO Luminograph (Japan).

### Immunoprecipitation assay

293 T cells were seeded in 6-well plates. Cells were transfected with 2 µg of pCAGGS EGFP, PEDV E (envelope), PEDV M (membrane), PEDV N (nucleocapsid) and PEDV S (spike) plasmids using PEI (12332A, Invitrogen). At 48 h post-transfection, the cells were washed in cold PBS, lysed with RIPA buffer for 30 min at 4 °C with protease inhibitor (Thermo Fisher, 88265), and then the supernatant was collected. Anti-c-myc was added to each tube and incubated overnight at 4 °C. Then, the AG beads (Santa Cruz, ab-2003) were incubated with the above samples overnight at 4 °C. After washing four times with RIPA buffer, the eluted samples were separated by SDS–PAGE on 12% gels under reducing conditions.

### Gene knockdown by siRNA

siRNAs obtained from GenePharma (Shanghai, China) were designed to bind endogenous HSP70 (GenBank Accession Number XM_007973082). Vero cells were seeded into 12-well plates, and the transfection mixture was prepared with 200 μL of Opti-MEM medium (Gibco, USA) containing Lipofectamine 3000 (Invitrogen, USA) and 200 μM of each siRNA (Table 2). At 48 h post-transfection, the cells were prepared for various experiments. The silencing efficiency was determined by Western blot assay.

**Table 2:** **siRNA used in this study.**

siRNA	Sequence (5′-3′)
#576 F	GCAACGUGCUCAUCUUUGATT
#576 R	UCAAAGAUGAGCACGUUGCTT
#1070 F	GACCUGAACAAGAGCAUCATT
#1070 R	UGAUGCUCUUGUUCAGGUCTT
scramble F	UUCUCCGAACGUGUCACGUTT
scramble R	ACGUGACACGUUCGGAGAATT

### Statistical analysis

Data are presented as the mean ± standard deviation (SD). Statistical significance was calculated using SPSS and GraphPad Prism 8. A *p *value < 0.05 was considered statistically significant. Asterisks in figures indicate statistical significance (*p < 0.05, **p < 0.01, ***p < 0.001).

## Results

### PEDV infection significantly increased HSP70 expression

**Figure 1:**
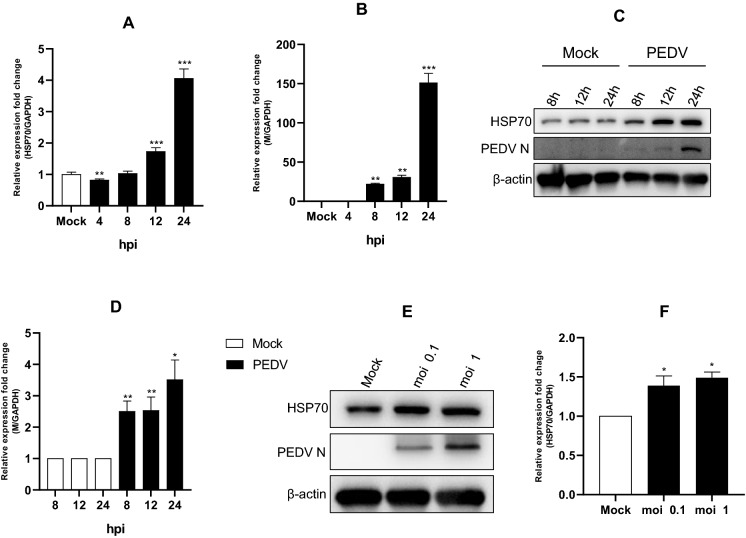
**Cellular HSP70 expression level in PEDV-infected cells. A**–**B** Vero cells were infected with PEDV at an MOI of 1 and harvested at different times as indicated. Total RNA was isolated to analyse HSP70 and viral M gene mRNA levels by quantitative RT–PCR. The mRNA levels of HSP70 and viral M were normalized to the mRNA levels of GAPDH. **C**–**D** Cell extracts were analysed by Western blot using anti-HSP70, anti-PEDV, and anti-β-actin antibodies. β-actin served as an internal control. The protein levels of HSP70 and PEDV were quantified by determining band intensities and normalized to β-actin. **E** IPEC-J2 cells were infected with PEDV at an MOI of 0.1 or 1. Cells were harvested at 24 hpi. The cell extracts were analysed by Western blot using anti-HSP70, anti-PEDV, and anti-β-actin antibodies. β-actin served as an internal control. **F** The levels of HSP70 and PEDV protein in IPEC-J2 cells were quantified by determining band intensities, and subsequent normalization to the levels of data is the mean ± SD (*n* = 3, *p < 0.05, **p < 0.01, ***p < 0.001).

We investigated the correlation between PEDV infection and HSP70 expression. PEDV-infected Vero cells induced significantly higher expression of HSP70 than mock-infected cells in a time-dependent manner (Figure 1A). We found that HSP70 expression was induced at 12 h post-infection (hpi), and its levels increased ~tenfold compared to the levels at 8 hpi. Shockingly, HSP70 expression increased by ~10 000-fold between 12 and 24 hpi. Based on these results, we concluded that PEDV infection strongly upregulates HSP70 expression. We also examined PEDV mRNA levels over time. We found that PEDV mRNA levels significantly increased from 8 hpi onwards, reaching a maximum at 24 hpi, which closely mirrored HSP70 activation (Figure 1B). We detected PEDV mRNA at 8 hpi and identified an ~70% increase between 12 and 18 hpi. These results were confirmed by Western blot. Western blot analysis confirmed that endogenous HSP70 expression was induced from 8 hpi (~2.5-fold higher than mock control), and there was a large increase between 8 and 12 hpi (Figure 1C). In addition, HSP70 expression increased by ~3.5-fold by 24 hpi. Taken together, our results clearly demonstrate that PEDV infection strongly upregulates endogenous HSP70 mRNA and protein expression. Additionally, we wanted to confirm our results in other PEDV-susceptible cell lines to ensure that the results were not specific to Vero cells. Vero cells are of monkey origin; thus, we wanted to confirm our findings in a swine cell line. We performed similar experiments using IPEC-J2 cells. Western blot analysis revealed similar findings in IPEC-J2 and Vero cells in relation to PEDV infection (Figure 1E). PEDV induced endogenous HSP70 expression in IPEC-J2 cells in an MOI-dependent manner. PEDV infection increased endogenous HSP70 expression levels by ~30–50% compared to mock-infected cells (Figure 1F).

### Colocalization of PEDV and HSP70

**Figure 2:**
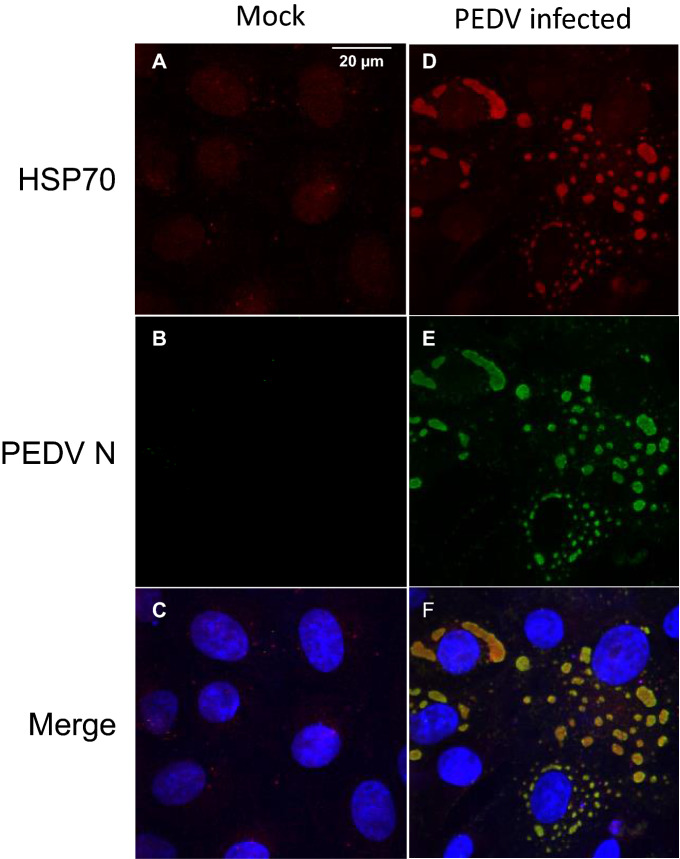
**Colocalization of HSP70 and PEDV.** Vero cells were infected with PEDV at an MOI of 1. Cells were fixed at 24 hpi, and double immunofluorescence analysis was performed with anti-PEDV (green) and anti-HSP70 (red) antibodies. Mock treatments served as negative controls. The nuclei were stained with Hoechst 333258 (blue). **A** Anti- HSP70 staining of mock-infected cells. **B** Anti-PEDV staining of mock-infected cells. **C** Merged image. **D** Anti- HSP70 staining of PEDV-infected cells. **E** Anti-PEDV staining of PEDV-infected cells. **F** Merged image.

Colocalization of HSP70 and PEDV was confirmed using confocal microscopy. In PEDV-infected Vero cells, PEDV was found only in the cytoplasm (Figure 2E). We confirmed the results using anti-PEDV N protein antibodies generated in mice. As shown in Figure 2D, HSP70 was also highly expressed in almost the same location as the PEDV protein. When we merged, PEDV proteins were expressed in exactly the same locations where HSP70 was expressed (Figure 2F). Therefore, we concluded that HSP70 and PEDV colocalized in PEDV-infected Vero cells.

### Conformation of correlation using HSP70 inhibitors and heat shock treatment

**Figure 3:**
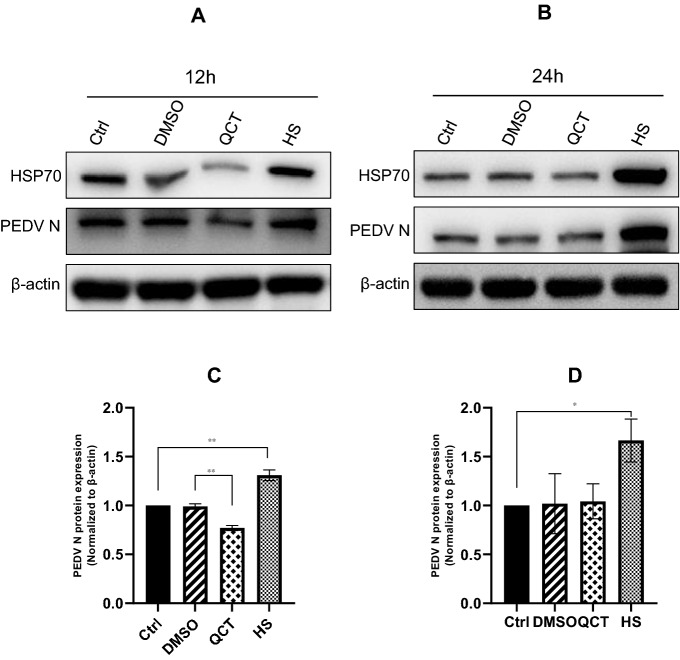
**Quercetin and heat shock treatment. A**–**D** Vero cells were heated (HS) or not (Ctrl) or treated with quercetin (100 μM; QCT) or not (DMSO). Twelve hours later, cells were inoculated with PEDV at an MOI of 1. Cells were harvested at 12 h and 24 h for Western blot analysis. The levels of PEDV N protein were quantified by determining band intensities, which were then normalized to the levels of β-actin. Data are the mean ± SD (*n* = 3, *p < 0.05, **p < 0.01, ***p < 0.001) **A** Cell lysates collected at 12 hpi were subjected to Western blot assay with anti-HSP70, anti-PEDV, and anti-β-actin antibodies. **B** Cell lysates collected at 24 hpi were subjected to Western blot assay with anti-HSP70, anti-PEDV, and anti-β-actin antibodies. **C**–**D** Quantification of 12 h and 24 h results.

Next, to confirm the role of HSP70 in PEDV replication, we used the HSP inhibitor quercetin as an HSP synthesis inhibitor and heat shock as an HSP70 activator. We compared HSP70 expression at 12 and 24 h after treatment. As shown in the Western blot data, quercetin treatment inhibited PEDV protein expression by ~27% (0.77 and 1.00, respectively) at 12 h (Figures 3A and C). In contrast, heat shock treatment (45 °C) resulted in a 31% increase in PEDV protein expression (1.00 and 1.31, respectively). However, there were no differences associated with quercetin treatment at 24 h (1.00 and 1.04) (Figures 3B and D). These results are similar to our preliminary data demonstrating that quercetin worked maximally at 12 h and had no effect afterwards (data not shown). In contrast, heat shock treatment increased HSP70 expression by approximately 67% at 24 h (1.00 and 1.67, respectively) (Figure 3B).

### Viral titre changes induced by HSP70 and heat shock treatment

**Figure 4:**
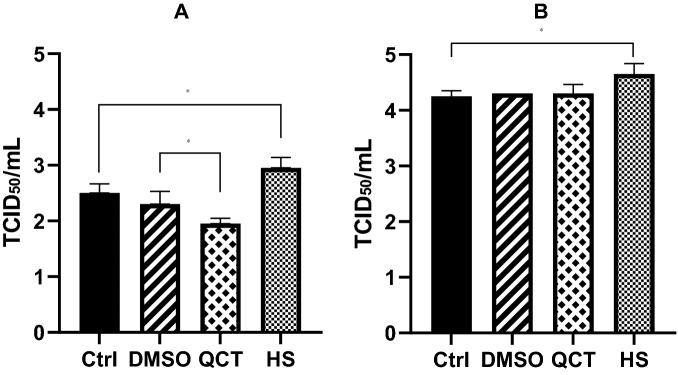
**The effects of quercetin and heat shock on viral titre.** Vero cells were heated (HS) or not (Ctrl) or treated with quercetin (100 μM; QCT) or not (DMSO). Twelve hours later, cells were inoculated with PEDV at an MOI of 1. The viral titers of cell supernatants were determined at 12 hpi and 24 hpi. Data are mean ± SD (*n* = 3, *p < 0.05). **A** Viral titre after 12 h. **B** Viral titre after 24 h.

We also compared viral replication by measuring viral titers after quercetin and heat shock treatment. There was a 22% decrease in PEDV titre following 12 h of quercetin treatment (TCID_50_ 2.5 and 1.95, respectively) (Figure 4A). In contrast, there was an 18% increase in PEDV titre following 12 h of heat shock treatment (TCID_50_ 2.5 and 2.99, respectively). However, there was no difference between the quercetin, DMSO, and negative control groups at 24 h (TCID_50_ 4.25, 4.30, and 4.30, respectively) (Figure 4B). However, there was still a 9.4% increase in PEDV titre following 24 h of heat shock treatment (TCID_50_ 4.25 and 4.65, respectively). PEDV protein levels also increased by ~2.5-fold following heat shock treatment but decreased by ~50% following quercetin treatment (Figure 4C). Therefore, we concluded that HSP70 expression significantly increased PEDV protein expression and resulted in the production of more progeny virions.

### The effect of knockdown or overexpression of HSP70 on PEDV replication

**Figure 5:**
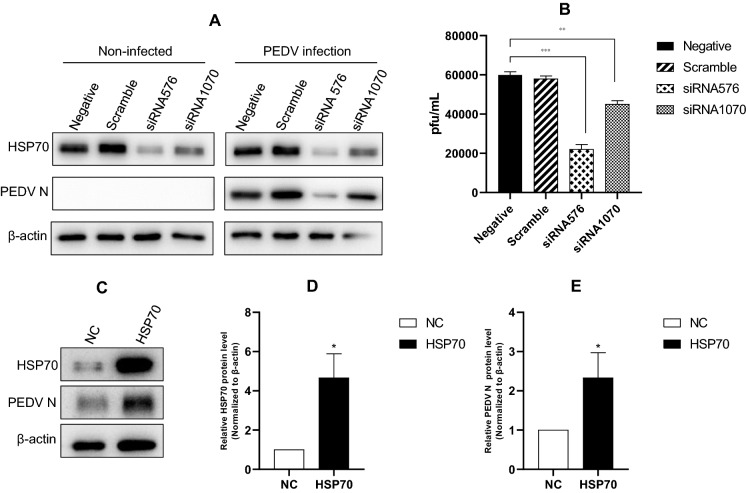
**Comparison of HSP70 and PEDV protein expression in HSP70 knockdown and overexpressed cells.** Vero cells were transfected with no siRNA (negative), scramble siRNA (scramble), or different siRNAs targeting HSP70 (siRNA576 and siRNA1070). Twenty-four hours later, the cells were mock-infected or infected with PEDV at an MOI of 1. **A** Western blot analysis of noninfected and PEDV-infected cells. **B** The viral titers of cell supernatants were determined 24 hpi. Data are mean ± SD (*n* = 3). **C** Vero cells were infected with a lentivirus control (NC) or pLenti-HSP70 (HSP70). Cells were harvested at 24 hpi for Western blot analysis. **D**–**E** The levels of HSP70 and PEDV protein were quantified by determining band intensities and subsequent normalization to the levels of β-actin. Data are the mean ± SD (*n* = 3, *p < 0.05, **p < 0.01, ***p < 0.001).

To confirm our findings, we performed HSP70 knockdown using siRNAs. Among the siRNAs tested, siRNA576 demonstrated the best results, and siRNA1070 produced reasonable results. In all knockdown experiments, scrambled siRNA was used as a negative control. The results showed that knockdown of HSP70 using siRNAs significantly reduced PEDV protein synthesis (Figure 5A). HSP70 knockdown resulted in an ~70% reduction in PEDV titre with siRNA576 and a 25% reduction with siRNA1070 (Figure 5B). No change was observed in the negative control. We then examined the Western blot results (Figure 5A) and found that these results were similar to what was observed in an HSP70-stable cell line. The results showed that PEDV protein expression was high in the HSP70-stable cell line (Figures 5C–E). These results strongly suggest that PEDV protein synthesis is closely related to HSP70 expression.

### The correlation between PEDV structural proteins and HSP70

**Figure 6:**
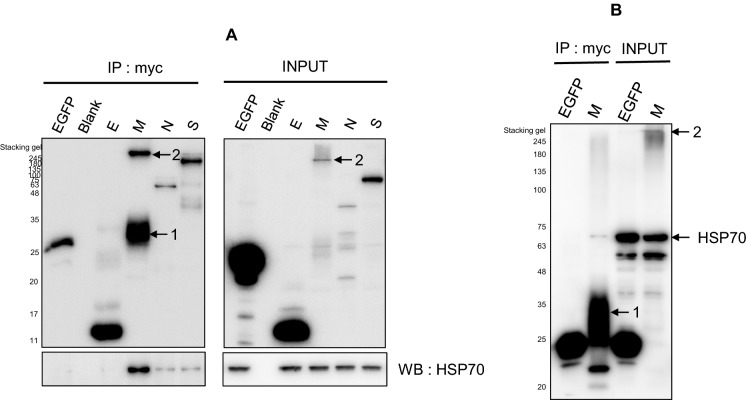
**Interaction between the PEDV M protein and HSP70.** Cells were transfected with PEDV structure proteins. After 48 h, the cells were harvested, and immunoprecipitation was performed. The transfected protein molecular weights were as follows: PEDV E (13 kDa), PEDV M (28 kDa), PEDV N (55 kDa) and PEDV S (150 kDa). **A** Immunoprecipitation assays demonstrated that endogenous HSP70 bound only to the PEDV M protein in transfected cells. PEDV M protein expressed in two forms, one in stacking gel (marked as 2 in Figure) and the other as approximately 28 kDa (marked as 1 in Figure). **B** As a form of validation, cells were transfected with HSP70 and PEDV M protein DNA only, and the analysis was performed again. All immunoprecipitation data clearly confirmed that immunoprecipitated PEDV M (number 1) was located at 28 kDa, and PEDV M (number 2) was located in stacking gels in the input data.

Next, we sought to determine which PEDV structural proteins are correlated with HSP70. We transfected each of the PEDV structural proteins (E, M, N and S) and performed immunoprecipitation (IP). As shown in Figure 6A, the PEDV M protein clearly immunoprecipitated with HSP70. PEDV M protein expressed in two forms, one in stacking gel (marked as 2 in Figure) and the other as approximately 28 kDa (marked as 1 in Figure). As shown by the IP results, only the PEDV M protein showed significant results, although there were very minor bands from the other structural proteins. As an expression control, we also showed whole-cell lysate images. To confirm this hypothesis, we performed IP experiments only with the M protein and found that the M protein clearly immunoprecipitated with HSP70, as shown in Figure 6B. Similar to Figure 6A, immunoprecipitated PEDV M (number 1) was located at 28 kDa, and PEDV M (number 2) was located in stacking gels in the input data.

### Colocalization of the PEDV M protein and HSP70

**Figure 7:**
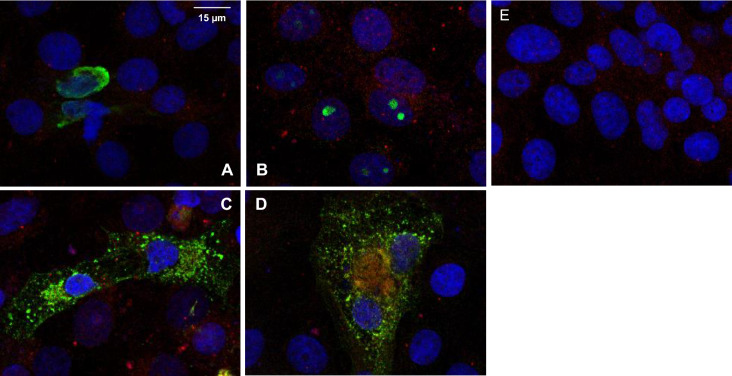
**Colocalization of PEDV M and HSP70.** Cells were transfected with PEDV structure proteins. After 48 h, IFA was performed with anti-Myc (green) and anti-HSP70 (red) antibodies. Nuclei were stained with Hoechst 33,258 (blue). Colocalization between PEDV M and HSP70 was confirmed by confocal microscopy. **A** pCAGGS PEDV N-myc, **B** pCAGGS PEDV S-myc, **C** pCAGGS PEDV E-myc, **D** pCAGGS PEDV M-myc, and **E** non-transfected control. All images are merged.

Colocalization of the PEDV M protein and HSP70 was confirmed using confocal microscopy. As shown by the IFA results, only the PEDV M protein clearly colocalized with HSP70 (Figure 7D). We could not detect colocalization of HSP70 with any PEDV structural proteins other than the M protein. Even though there were very minor bands in the IPs with other structural proteins (Figure 6A), confocal microscopy clearly confirmed the colocalization of HSP70 and the PEDV M protein. Based on these results, we concluded that the PEDV M protein directly interacts with HSP70, and this interaction activates both HSP70 expression and PEDV replication.

### Specificity of the correlation between the PEDV M protein and HSP70

**Figure 8:**
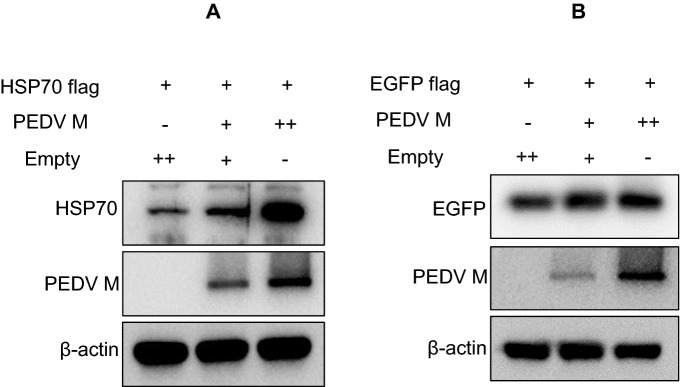
**Specificity of the correlation between PEDV M and HSP70 expression.** Cells were transfected with 1 or 2 µg of PEDV M DNA together with either HSP70 or EGFP plasmids. After 48 h, cells were harvested and subjected to Western blot analysis. **A** Cells were cotransfected with 1 or 2 µg of PEDV M DNA together with HSP70 and subjected to Western blot analysis. **B** Cells were cotransfected with 1 or 2 µg of PEDV M DNA together with EGFP and subjected to Western blot analysis.

Next, we examined the specificity of the correlation between HSP70 and the PEDV M protein. First, we monitored changes in HSP70 expression in response to various amounts of the PEDV M protein. We transfected different amounts (1 µg and 2 µg) of the M protein plasmid pCAGGS-PEDV-M together with either HSP70 or EGFP plasmids. In Western blot results, transfection of 2 µg of PEDV M DNA resulted in higher HSP70 expression than in cells transfected with only 1 µg (Figure 8A). Actin-normalized data revealed that PEDV M expression increased ~threefold compared to the empty vector-transfected control (Figure 8A). Interestingly, in the same EGFP overexpression experiments, even with increased PEDV M, there was no change in the level of EGFP expression (Figure 8B). Based on these results, we concluded that PEDV M specifically interacts with HSP70 in a dose-dependent manner.

## Discussion

Many positive-stranded RNA viruses produce specific proteins that interact with chaperones in infected cells. The role of these proteins relates to the folding of abundant viral proteins, regulation of viral replication via activation of replication proteins, and reduction of host antiviral responses. Among these host chaperones, HSP70 has been reported to be most frequently involved in viral replication. Changes in HSP70 expression induced by viral infection control intracellular defence mechanisms. For example, in influenza A virus, the ribonucleoprotein is interrupted by HSP70, which blocks its replication in vitro and in vivo [17]. HIV-1 viral protein R (Vpr) is related to G2 cell cycle arrest. HSP70 counteracts Vpr G2 growth arrest, which is positively regulated by HIV-1 replication [16]. Rotavirus (RF strain) viral protein synthesis and progeny virus production were increased by HSP70 silencing in Caco-2 cells. HSP70 silencing reduced the ubiquitination of rotavirus structural proteins [33]. Many viruses utilize HSP70 for their replication. In porcine reproductive and respiratory syndrome virus, upregulation of HSP70 in MARC-145 cells significantly increased viral protein levels and virion production. In classical swine fever virus, NS5A directly interacts with HSP70 and promotes HCV replication [34, 35]. In classical swine fever virus, NS5A directly interacts with HSP70, an interaction that plays a pivotal role in the viral life cycle [36]. In tomato bushy stunt virus, the replicase complex requires HSP70, which plays a role in the assembly of viral replicase [37]. In dengue virus infection, downregulation of HSP70 in THP-1 cells significantly decreased viral protein and virion production levels [38]. Kong et al. reported that HSP70 was upregulated in the intestine in both cold temperature-exposed pigs and cold-exposed Vero cells. They concluded that HSP70 positively regulated PEDV mRNA synthesis and protein expression. These results are similar to our results in the correlation between HSP70 and PEDV replication [39].

In the present study, we examined the correlation between PEDV and HSP70. We found that HSP70 plays a very important role in PEDV replication. PEDV infection induced the expression of HSP70, and as a result, PEDV replicated in high numbers and eventually generated much higher progeny virions (Figure 1). As expected, heat shock treatment induced higher levels of viral proteins and viral replication. In contrast, quercetin treatment significantly decreased the levels of viral proteins and viral replication (Figures 3 and 4). Knockdown of HSP70 by siRNAs clearly confirmed that inhibition of HSP70 reduces PEDV protein expression and viral replication (Figures 5A and B). Conversely, overexpression of HSP70 increased PEDV protein expression and viral replication (Figures 5C and D). In conclusion, HSP70 plays a very important role in PEDV replication. Although we were not able to completely block HSP70 expression (too many cellular HSP70 molecules in many different cellular locations), our results are still meaningful.

The PEDV M protein is a membrane protein that is wrapped around viral particles. Previous studies have identified that the PEDV M protein plays an important role in viral assembly [40]. Additionally, the PEDV M protein affects cell growth, cell cycle progression, and interleukin 8 (IL-8) expression. Expression of the PEDV M protein in an intestinal epithelial cell line arrested the cell cycle at S-phase via the cyclin A pathway. However, the PEDV M protein does not induce endoplasmic reticulum stress and does not activate NF-κB [41]. In addition to functioning in virion formation and cell cycle arrest, the PEDV M protein also reduces host cell immune responses. Recent LC–MS/MS studies have reported several potential binding partners of the PEDV M protein [42].

In this study, we detected a direct interaction between the PEDV M protein and HSP70. We found that PEDV M and HSP70 colocalized and coimmunoprecipitated (Figures 6 and 7). This correlation between M and HSP70 occurred in a dose-dependent manner (Figure 8). It was not Vero cell specific because the correlation was also confirmed in IPEC-J2 cells (Figure 1).

Further studies examining the interaction between HSP70 and non-structural PEDV proteins are required. In summary, we demonstrated that HSP70 plays a very important role in PEDV replication. PEDV infection induced HSP70 expression, which boosted PEDV protein expression and the production of progeny virions. While the PEDV M protein directly interacts with HSP70 and promotes PEDV replication, the mechanism through which HSP70 promotes PEDV replication is unclear. Further mechanism study is needed.
